# Dysphagia, voice problems, and pain in head and neck cancer patients

**DOI:** 10.1007/s00405-020-06584-6

**Published:** 2021-01-16

**Authors:** Veit Zebralla, Gunnar Wichmann, Markus Pirlich, Carola Hammermüller, Thomas Berger, Klara Zimmermann, Thomas Neumuth, Anja Mehnert-Theuerkauf, Andreas Dietz, Andreas Hinz, Susanne Wiegand

**Affiliations:** 1 Department of Otolaryngology, Head and Neck Surgery, Clinic of Otolaryngology, Head and Neck Surgery, University of Leipzig, Leipzig, Germany; 2 Department of Medical Psychology and Medical Sociology, University of Leipzig, Leipzig, Germany; 3 Innovation Center Computer Assisted Surgery (ICCAS), University of Leipzig, Leipzig, Germany

**Keywords:** Dysphagia, Voice impairment, Cancer pain, Head and neck cancer, Symptom burden

## Abstract

**Purpose:**

Head and neck cancer (HNC) and its treatment can leave devastating side effects with a relevant impact on physical and emotional quality of life (QoL) of HNC patients. The objectives were to examine the amount of dysphagia, voice problems, and pain in HNC patients, the impact of sociodemographic, behavioral, and clinical factors on these symptoms, the psychometric properties of the EAT-10, and the relationship between these symptoms and QoL variables.

**Methods:**

HNC patients attending for regular follow-up from 07/2013 to 09/2019 completed questionnaires (Eating Assessment Tool-10 (EAT-10); questions from the EORTC QLQ-C30 and EORTC H&N35) on dysphagia, voice problems, pain, fatigue, and QoL collected with the software OncoFunction. Associations between prognostic factors and symptoms were tested with analyses of variance (ANOVAs). Associations between the symptom scales and QoL variables were expressed with Pearson correlations.

**Results:**

Of 689 patients, 54.9% suffered from dysphagia, the EAT-10 proved to be a reliable measure. The mean voice score was 37.6 (± 33.9) [range 0–100], the mean pain score 1.98 (± 2.24) [range 0–10]. Trimodality treatment was associated with the highest dysphagia scores. Dysphagia, voice problems, and pain significantly correlated with each other, the highest association was found for dysphagia and pain (*r* = 0.51). QoL was strongly correlated with dysphagia and pain (*r* = − 0.39 and *r* = − 0.40, respectively), while the association with voice problems was weaker (*r* = − 0.28).

**Conclusion:**

Dysphagia is an important symptom in HNC patients greatly affecting patients’ QoL and significantly correlating with voice problems and pain.

## Introduction

In the last decades, improvements in diagnostic technologies and advancements in surgery, radiation, chemo- and immunotherapy led to improved local tumor control and lower mortality rates in head and neck cancer (HNC) patients [1]. The number of HNC survivors additionally increased due to the changing epidemiology of the disease, most notably the increase in HPV-positive oropharynx carcinoma [2]. However, the disease and its treatment can leave devastating side effects with a relevant impact on the physical and emotional wellbeing of HNC patients. Therefore, there is a high need for supportive care among HNC survivors, who may live with significant symptom burden for a long time. In 2013, we, therefore, introduced a software (OncoFunction) at our hospital to assess patient-reported outcome and monitor HNC patients [3]. Prior studies among HNC survivors indicate high prevalence of pain, dysphagia, and voice problems [4–8]. Dysphagia impacts nutrition and hydration can deteriorate patient`s social contacts and significantly diminish the quality of life (QoL). Dysphagia represents an independent risk factor for worse survival [9]. One frequently used instrument for measuring dysphagia is the Eating Assessment Tool (EAT-10) [10]. It proved to be a valid and reliable instrument in groups of patients who were treated for dysphagia. In our study, we intend to test the psychometric properties of the EAT-10 in a large German sample of HNC patients. Pain is also a frequent symptom of HNC patients leading to physical and psychological impairment. The prevalence of pain at HNC diagnosis is estimated to be 40–85% [6–8]. The reasons are multifactorial, but the main cause is considered to be the rich nerve supply of the head and neck area. Pain has been demonstrated to be underreported by patients and often not adequately assessed by physicians resulting in non-adequate pain management [11]. Impaired voice quality and speech are typical sequels of HNC and its treatment. Not only surgery of the larynx can lead to voice problems, surgery of oral and pharyngeal cancer can also affect articulation and speech. (Chemo)-radiation may lead to impaired vocal fold vibration with incomplete closure, muscle atrophy, dryness of the mucosa, fibrosis, and edema [12]. In summary, poor voice quality and/or aspiration can be described as a non-functional larynx after treatment of HNC. For these reasons, HNC and its treatment have a relevant impact on health-related QoL. So far, most of the studies analyzing symptoms after HNC have investigated only very small case numbers and studies assessing patient-reported dysphagia and voice impairment in HNC are rare [13, 14].

The objectives of this study were (a) to explore the amount of dysphagia, voice problems, and pain in a large cohort of HNC patients, (b) to examine the impact of sociodemographic, behavioral, and clinical factors on these symptoms, (c) to test the psychometric properties of the EAT-10, and (d) to examine the relationship between these symptoms and QoL variables.

## Methods

### Patients

The sample comprised HNC patients who attended the outpatient clinic for head and neck cancer of a University hospital for regular follow-up appointments from July 2013 to September 2019. Inclusion criteria were diagnosis of HNC and age older than 18; patients with severe cognitive impairment, patients who refused participation, and those who were unable to read or complete the questionnaires on the tablet computer were excluded.

### Data collection

The data were collected with the software OncoFunction basing on the International Classification of Function [15]. The used screening tool is recommended by the German Cancer Society. OncoFunction is available on tablet computers with a touch screen. All participants signed informed consent digitally. Patients filled in OncoFunction before the follow-up visit with their physician started. The results were available and visible in real-time in a program, which is linked to the hospital information system. Based on the results, the physician could identify and support patients’ problems. Additionally, the ECOG status and the BMI were documented by the physician. The usability of the system has been demonstrated before [3]. Since patients visited the outpatient clinic at different stages of their follow-up, data were collected at different time points ranging from 3 months to greater than 5 years after initial treatment of HNC.

### Instruments

The Eating Assessment Tool-10 (EAT-10) is a validated, symptom-specific self-assessment instrument designed to rate the patient´s perception of their swallowing impairment [10]. It consists of ten items. Each of the 10 items is rated by the patient on a 5-level scale ranging from 0 (no impairment) to 4 (a severe problem), with a total score range of 0–40. A score of 3 or greater is considered abnormal and indicative of clinically significant dysphagia. The EAT-10 is frequently used in clinical practice and research as a screening tool for patients with suspected swallowing problems [16–18] and has also been validated in a German HNC sample by Zaretsky et al. [19].

For measuring voice problems, we used two questions of the questionnaire EORTC QLQ-H&N35 [20]. The respondents were asked to state to what degree they experienced problems when talking to other people or talking on the telephone. A four-point scale is used for scoring the responses: 1 (not at all); 2 (a little); 3 (quite a bit) and 4 (very much). According to the methodology of the EORTC QLQ-C30, the scores are transformed into the range 0–100.

Pain was assessed with a single item, ranging from 0 (no pain a t all) to 10 (maximum possible pain).

The EORTC QLQ-C30 is one of the most frequently used instruments to analyze health-related QoL [21]. It includes a three-item fatigue subscale (“Did you need to rest?”; Have you felt weak”; “Were you tired?”) with four answer options: 1 (not at all); 2 (a little); 3 (quite a bit) and 4 (very much). The global health status/quality of life subscale from EORTC QLQ-C30 is composed of two items asking for the overall health rate and the overall quality of life during the past week. The items are rated by the patients on a 7-level scale ranging from 1 (very poor) to 7 (excellent). The fatigue score and the global health/QoL score are also transformed into the range 0–100.

### Statistical analyses

For the EAT-10, we calculated part-whole-corrected item-test-correlations and Cronbach’s alpha for measuring internal consistency. A confirmatory factor analysis (CFA) was calculated to test the model fit of the one-dimensional model of the EAT-10. We used the coefficients CFI, TLI, RMSEA, and SRMR.

The associations between (behavioral and clinical) prognostic factors and the symptoms were statistically tested with analyses of variance (ANOVAs) including age and sex as covariates.

Associations between the symptom scales and quality of life variables were expressed with Pearson correlations. The CFA was calculated with MPlus, all other analyses were performed with SPSS, version 20.

## Results

### Sample characteristics

Among the 1026 patients who were eligible for the study, 710 completed the EAT-10, 860 completed the Voice scale, and 871 the Pain scale. We restricted the analyses to those patients who completed all of these three questionnaires, which resulted in a sample of 689 patients. Table 1 shows the characteristics of this sample.

**Table 1:** Patient characteristics

	Total (*n* = 689)		Males (*n* = 540)		Females (*n* = 149)	
	*N*	%	*N*	%	*N*	%
Age group						
18–59 y	275	39.9	210	38.9	65	43.6
60–69 y	238	34.5	200	37.0	38	25.5
≥ 70 y	176	25.5	130	24.1	46	30.9
Occupation						
Not occupied	513	74.5	412	76.3	101	67.8
Occupied	176	25.5	128	23.7	48	32.2
Alcohol drinking						
No	484	70.2	349	64.6	135	90.6
Yes	205	29.8	191	35.4	14	9.4
Smoking						
No	503	73.0	379	70.2	124	83.2
Yes	186	27.0	161	29.8	25	16.8
Tumor group						
Oral cavity	102	14.8	77	14.3	25	16.8
Oropharynx	229	33.2	175	32.4	54	36.2
Larynx, hypopharynx	214	31.1	194	35.9	20	13.4
Other	144	20.9	94	17.4	50	33.6
Tumor stage*						
I	130	21.1	100	20.4	30	23.4
II	97	15.7	78	16.0	19	14.8
III	98	15.9	71	14.5	27	21.1
IV	292	47.3	240	49.1	52	40.6
Treatment group						
1: OP + RT−CT−	202	29.3	152	28.1	50	33.6
2: OP + RT + CT−	166	24.1	133	24.6	33	22.1
3: OP + RT + CT +	194	28.2	154	28.5	40	26.8
4: OP−RT + CT +	93	13.5	78	14.4	15	10.1
5: Other	34	4.9	23	4.3	11	7.4
Metastases						
No	385	55.9	300	55.6	85	57.0
Yes	304	44.1	240	44.4	64	43.0
Tracheostomy*						
No	562	81.6	429	79.4	133	89.3
Yes	125	18.1	109	20.2	16	10.7
Feeding tube						
No	582	84.5	450	83.3	132	88.6
Yes	107	15.5	90	16.7	17	11.4
ECOG performance*						
0	195	39.2	150	38.4	45	42.5
1	262	52.7	210	53.7	52	49.1
2–4	40	8.0	31	7.9	9	8.5
Body mass index						
≤ 20 kg/m^2^	92	13.4	59	10.9	33	22.1
20 – ≤ 25 kg/m^2^	328	47.6	267	49.4	61	40.9
25 – ≤ 30 kg/m^2^	195	28.3	162	30.0	33	22.1
> 30 kg/m^2^	74	10.7	52	9.6	22	14.8
Time since diagnosis*						
≤ 9 months	342	49.8	260	48.2	82	55.4
> 9 months	345	50.2	279	51.8	66	44.6

*OP + , OP−* surgery yes/no, *RT + , RT−* radiotherapy yes/no, *CT + , CT−* chemotherapy yes/no

*Missing data not reported

Table 2 shows the item characteristics of the EAT-10. All items positively contributed to the sum score, all *r*_it_ coefficients were greater than 0.60. The internal reliability (alpha = 0.94) was very good.

**Table 2:** EAT. Mean scores, item–test correlations for the cancer patients

Item		*M*	SD	*r*_*it*_	Alpha (del)
1	Weight loss	0.73	1.14	0.70	0.93
2	Problems with meals	1.06	1.43	0.80	0.93
3	Swallowing liquids	0.49	0.94	0.70	0.93
4	Swallowing solids	1.10	1.31	0.84	0.92
5	Swallowing tablets	0.81	1.23	0.74	0.93
6	Swallowing painful	0.55	0.97	0.73	0.93
7	Reduced pleasure to eat	0.96	1.31	0.85	0.92
8	Food sticks in throat	0.42	0.90	0.62	0.93
9	Coughing when eating	0.65	1.06	0.64	0.93
10	Swallowing stressful	0.82	1.13	0.86	0.92
Sum	(range 0–40)	7.62	9.15	–	alpha = 0.94

*r*_*it*_ part-whole corrected item–test correlation, *alpha (del)* Cronbach’s alpha if item deleted

### Psychometric properties of the scales

The CFA results of the one-dimensional EAT-10 model resulted in the following coefficients: Chi^2^ (DF) = 578.124 (35), CFI = 0.890, TLI = 0.858, RMSEA = 0.153, and SRMR = 0.055.

Using the EAT-10 cutoff ≥ 3 for abnormal dysphagia, 378 of the patients (54.9%) suffered from dysphagia. 217 patients (31.5%) had a score of 0 (no dysphagia at all), and 223 patients (32.4%) showed scores ≥ 10. 125 patients (18.1%) needed a tracheotomy and 107 (15.5%) required a feeding tube to ensure nutrition.

The voice scale yielded the coefficients *M* (Mean) = 37.6, SD = 33.9, and Cronbach’s alpha = 0.93. A proportion of 35.1% (*n* = 242) of the patients had no voice problems at all (score = 0), and 8.6% of the patients (*n* = 59) reached the maximum score of 100 on the voice scale.

The mean score and the SD of the pain scale (range 0–10) were *M* = 1.98 and SD = 2.24. Of the 689 patients, 277 patients (40.2%) were completely free from pain with a score of 0, and 162 patients (23.5%) reported a pain score of 4 or above.

### Associations between sociodemographic, behavioral, and clinical factors and dysphagia, voice problems, and pain

Table 3 presents the mean symptom scores for subgroups of the sample which were defined by sociodemographic, behavioral, and clinical variables. Females reported slightly higher pain levels (*M* = 2.3) than males (*M* = 1.9), while there were no significant gender effects in dysphagia and voice problems. The oldest age group (≥ 70 years) was characterized by the lowest burden in the three symptoms, the differences in dysphagia and pain were statistically significant.

**Table 3:** Mean scores depending on sociodemographic and clinical variables

	*n*	Dysphagia		Voice problems		Pain	
		*M*	SD	*M*	SD	*M*	SD
Sex		*p* = 0.826		*p* = 0.638		*p* = 0.030	
Males	540	7.7	9.3	38.0	33.2	1.9	2.2
Females	149	7.7	9.0	36.1	36.3	2.3	2.4
Age group		*p* = 0.006		*p* = 0.265		*p* = 0.011	
≤ 59 years	275	8.0	9.2	38.7	33.5	2.2	2.2
60–69 years	238	8.7	9.6	39.0	32.8	2.0	2.2
≥ 70 years	176	5.9	8.6	33.9	35.9	1.6	2.3
Occupational status		*p* < 0.001		*p* = 0.001		*p* = 0.015	
Not occupied	513	8.3	9.6	39.4	34.6	2.0	2.3
Occupied	176	5.8	7.7	32.2	31.2	1.9	2.0
Alcohol drinking		*p* < 0.131		*p* = 0.477		*p* = 0.565	
No	484	8.0	9.4	37.9	34.4	2.0	2.3
Yes	205	7.1	8.8	36.8	32.8	1.9	2.2
Smoking		*p* = 0.336		*p* = 0.414		*p* = 0.299	
No	503	7.3	8.7	42.5	35.6	2.1	2.3
Yes	186	8.5	10.2	40.5	34.5	2.3	2.5
Tumor localization		*p* < 0.001		*p* < 0.001		*p* = 0.015	
Oral cavity	102	6.8	8.9	35.1	31.5	2.3	2.3
Oropharynx	229	10.7	9.5	35.6	32.8	2.3	2.3
Larynx, hypopharynx	214	6.6	9.0	49.8	33.4	1.5	2.0
Other	144	5.1	8.1	24.3	32.3	1.9	2.3
Tumor stage		*p* < 0.001		*p* = 0.346		*p* = 0.095	
I	130	3.8	7.1	33.7	34.2	1.5	2.0
II	97	7.0	9.4	38.5	33.0	2.1	2.3
III	98	8.4	8.9	36.1	31.4	2.0	2.2
IV	292	9.7	9.7	40.4	34.3	2.0	2.3
Treatment group		*p* < 0.001		*p* = 0.297		*p* = 0.267	
1: OP + RT−CT−	202	4.7	7.9	35.7	35.1	1.7	2.1
2: OP + RT + CT−	166	7.3	9.0	39.5	34.4	2.0	2.3
3: OP + RT + CT +	194	9.9	9.1	39.4	31.9	2.3	2.3
4: OP−RT + CT +	93	9.8	10.5	32.6	34.0	1.9	2.1
5: Other	34	9.1	10.0	42.2	35.1	1.9	2.5
Metastases		*p* < 0.001		*p* = 0.617		*p* = 0.627	
No	385	6.2	8.9	36.8	33.8	1.9	2.3
Yes	304	9.6	9.3	38.5	34.0	2.1	2.2
ECOG performance		*p* < 0.001		*p* < 0.001		*p* < 0.001	
0	195	4.3	6.2	30.8	33.0	1.4	1.9
1	262	9.0	9.4	39.9	32.4	2.1	2.2
2–4	40	15.3	11.5	50.8	36.2	3.2	2.8
Body mass index		*p* < 0.001		*p* = 0.846		*p* = 0.025	
≤ 20 kg/m^2^	92	11.2	10.4	40.4	32.3	2.6	2.5
20 – ≤ 25 kg/m^2^	328	8.5	9.6	36.7	34.2	2.0	2.3
25 – ≤ 30 kg/m^2^	195	5.5	7.6	37.2	33.2	1.7	2.1
> 30 kg/m^2^	74	5.3	7.8	38.8	36.6	1.6	2.0
Time since diagnosis		*p* = 0.332		*p* = 0.222		*p* = 0.179	
≤ 9 months	342	8.1	9.1	39.3	34.5	2.1	2.3
> 9 months	345	7.3	9.4	35.9	33.4	1.8	2.2

*OP + , OP−* surgery yes/no, *RT + , RT−* radiotherapy yes/no, *CT + , CT−* chemotherapy yes/no

With respect to behavioural factors, like alcohol drinking and smoking, there were no significant differences regarding pain, voice problems and dysphagia. Occupied patients reported significantly less pain, dysphagia and voice problems than those who were not occupied.

Tumor localization had a significant impact on all three symptoms while tumor stage and presence of metastases had a significant effect on the EAT-10 scores but not on the pain and voice scale. Patients with small HNC had significantly less dysphagia than those with advanced ones. Oropharynx cancer led to the highest dysphagia scores while larynx and hypopharynx tumors resulted in significantly more voice problems. Patients with oral and oropharynx cancer reported significantly more pain than patients with HNC of other localizations.

Trimodality treatment was associated with the highest dysphagia scores, chemoradiation resulted in slightly lower scores, the lowest scores had patients who were only treated by surgery. There were no significant correlations between voice problems and pain with treatment modality. Worse Eastern Cooperative Oncology Group performance status (ECOG) scores (2–4) were correlated to the highest burden in the three symptoms.

Patients with BMI < 20 showed significantly higher values in EAT-10 and pain while obese patients reported lower values. Duration of post-therapeutic follow up did not reach any significance.

### Frequency of dysphagia

**Fig. 1:**
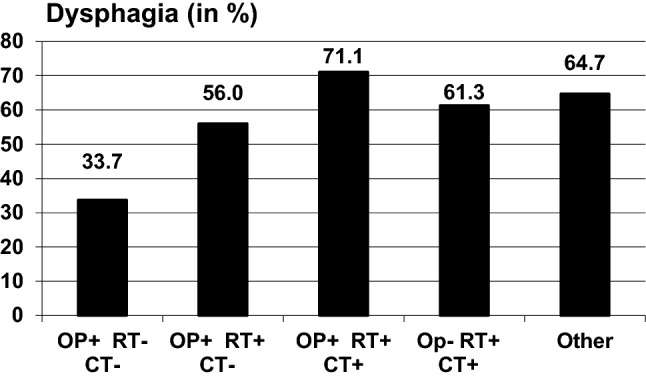
Frequency of dysphagia in relation to the treatment

**Fig. 2:**
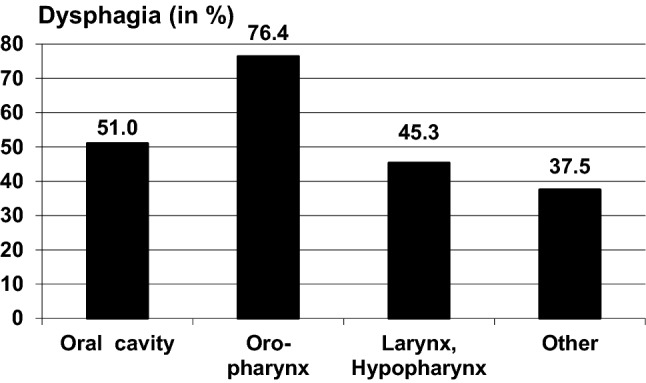
Frequency of dysphagia in relation to tumor localization

Using the criterion for dysphagia given by the authors of the EAT (score ≥ 3), 54.9% of the sample suffered from dysphagia. The frequency of dysphagia in relation to the tumor type and treatment is presented in Figs. 1 and 2. Patients with oropharynx cancer had the highest level of dysphagia, while patients receiving surgery but neither RT nor CT showed the lowest degree of dysphagia.

### Correlations between the scales

The correlations between the three symptom scales and the two additional scales fatigue and QoL of the EORTC QLQ-C30 are given in Table 4. All correlations are statistically significant with *p* < 0.001. The highest association was found for the relationship between dysphagia and pain (*r* = 0.51). QoL was strongly correlated with dysphagia and pain (*r* = − 0.39 and *r* = − 0.40, respectively), while the association with voice problems was somewhat weaker (*r* = − 0.28).

**Table 4:** Correlations among the scales

	Dysphagia	Voice problems	Pain
Dysphagia	–	–	–
Voice problems	0.25	–	–
Pain	0.51	0.22	–
Fatigue	0.45	0.38	0.46
Quality of life	− 0.39	− 0.28	− 0.40

## Discussion

The first aim of the study was to assess the burden of specific symptoms experienced by the HNC patients. About half of the patients (54.9%) suffered from dysphagia. The tracheostomy and feeding tube rates show that aspiration and the associated protection of the respiratory tract and securing of nutrition are relevant problems after therapy of HNC. When examining swallowing function, both fiber optic endoscopic evaluation of swallowing (FEES) and videofluoroscopy are considered to be the gold standard in dysphagia assessment [22]. We did not use objective swallowing assessments in this study but the EAT-10 has been shown to correlate in HNC patients with objective swallowing assessments like videofluoroscopy and FEES [17, 23], and the advantage of a patient survey is to assess how patients feel they are impacted by their swallowing dysfunction. Additionally, a screening tool allows the economical use of the FEES. Other studies also demonstrated dysphagia as a predominant side effect of HNC and its treatment [24]. For voice problems and the pain scale, there are no cut-off scores and no normative values. However, the mean score of 37.6 on the 0–100 scale for voice problems indicates that a substantial proportion of patients had voice problems.

The mean pain score of *M* = 1.98 (scale range 0–100) seems to be a hint that pain is not the predominant phenomenon in our patient cohort although HNC is characterized by a significantly greater pain perception compared to other tumors [25]. Because of the absence of normative scores, it is difficult to compare these scale means. The EORTC QLQ-C30 includes a pain scale (range 0–100), and there are normative values for this questionnaire. The mean pain score of the general population is 16.5; applied to the age and gender distribution of our HNC patient sample, the mean score is 19.6 [26], which is nearly identical with the mean of 19.8 which corresponds with the mean of 1.98 on the 0–10 pain scale. A reason for the low pain level in our cohort may be that these patients finished treatment on average 5 months before and had already experienced more pain. In other studies, pain was analyzed at the time of or directly after treatment [25]. In a study of 93 HNC patients, it could be shown that the incidence of pain dropped from 48% at diagnosis to 25% one year later [6].

The EAT-10 proved to be a reliable measure. The internal consistency was very good (alpha = 0.94). Other studies also found such excellent reliability coefficients [10, 19, 27]. Nevertheless, the results of the CFA showed that the model fit was not perfect. This means that the ten items of the test cannot be considered as being independent of one another except their common variance with the latent variable. Table 2 shows that the items “problems with food” and “swallowing solids” were mentioned most often. The EAT-10 is a homogenous test, with high correlations among all of the items. It might be useful to investigate whether a shortened version of the test is also be sufficient for assessing swallowing problems.

Concerning gender, there were no significant differences in two of the three symptom scales (dysphagia and voice problems) between males and females. Females reported slightly more pain than males. However, concerning age, there were more pronounced differences between the groups: older patients (70 years and above) reported the lowest burden. Since there are no normative scores for the symptom scales, based on samples of the general population, we cannot estimate to what degree these age differences are due to normal age affects, and to what degree they are HNC-specific. The quality of life questionnaire EORTC QLQ-C30 includes eight symptom scales. For these scales, there are normative scores [26, 28], and a general decline with increasing age cannot be observed for these scales. The pain scale of this questionnaire even shows a systematic increase with increasing age. Therefore, we assume that this age effect (reduced symptoms with increasing age) cannot be due to a general age effect.

Alcohol consumption and smoking were not significantly associated with the symptoms. Here it is impossible to conclude a causal relationship. Alcohol drinking and smoking may result in health problems, but it is also possible that patients with severe health problems decided to give up smoking and drinking. Longitudinal studies are necessary to explore the causality in this patient group.

All measured patient-reported outcomes were associated with employment status. It is important to improve these symptoms since many HNC patients are not of retirement age and could work for many more years. In the process of return to work and resuming everyday life, dysphagia, pain, and voice problems represent great barriers which were also demonstrated by previous studies [29, 30].

Analyzing tumor site, we found significant differences between hypopharynx/larynx cancer against oral cavity/oropharynx location with significantly better results in terms of voice problems for the latter but less pain in larynx/hypopharynx cancer. Considering tumor stage, as anticipated and demonstrated in previous studies, there was significantly more dysphagia in advanced tumor stages, on the other hand, no significant differences regarding voice problems and pain were noted. This is surprising at first sight, but there was a tendency towards better voice and less pain with smaller tumor stage. Patients affected by carcinomas of the hypopharynx/larynx and oral cavity showed a better swallowing function in comparison to those affected by oropharynx cancers who also had the highest frequency of dysphagia. Similar results could be shown in a previous study by Carmignani et al. [24], while other authors have found the opposite [31]. In our cohort, higher EAT-10 scores were moreover significantly related to metastatic disease.

When treatment strategy was considered, dysphagia has the highest incidence (71.1%) and was significantly worse in patients undergoing trimodality treatment (surgery + RT + CT), the group undergoing exclusive surgery had the lowest frequency of dysphagia (33.7%) and the lowest scores in the EAT-10. Voice problems and pain were not significantly correlated with treatment modality but there was a tendency for higher scores in the trimodality and surgery + radiation group. Existing literature on the correlation between treatment modality and symptoms has been inconclusive [14, 24]. As a potential reason for these findings, the need for only a single modality treatment in lower tumor stages can be discussed. In all, patients with worse ECOG performance status scores had the highest burden in the three symptoms dysphagia, voice impairment, and pain.

Concerning body weight, patients with body mass index ≤ 20 kg/m^2^ had the highest scores in the EAT-10 which seems logical since swallowing difficulties without sufficient supplementation lead to weight loss. The patients are in a vicious circle because malnutrition, in turn, leads to decreased activity, inducing more weight loss and lethargy [32, 33].

Previous studies suggest that swallowing disturbances, voice impairments, and pain have a major impact on QoL. This is confirmed by our data. Dysphagia, voice problems, and pain were associated with QoL. The impact of dysphagia (*r* = − 0.39) was nearly as high as that of pain (*r* = − 0.40), while the association between voice problems and QoL was somewhat weaker (*r* = − 0.28). This underlines the importance of dysphagia for the total assessment of QoL in HNC patients. Cramer et al. [34] analyzed pain among HNC survivors and showed a high incidence of pain that correlated with worse overall QOL across multiple specific domains. Moreover, a relationship between physical impairment and compromised psychosocial functions has been reported in the literature [35].

Regardless of QoL, the assessed symptoms are important for patient’s prognosis. Previous studies in HNC patients showed that the presence of pain and dysphagia at the time of diagnosis is associated with impaired survival [36–40]. In a prospective cross-sectional cohort study, it could be shown that patient-reported dysphagia was the most effective predictor of disease-specific survival and also predicted disease recurrence [36].

### Limitations

Our study is limited by its cross-sectional design. Patients were surveyed at different times in their treatment course; however, there were no significant disparities with regard to the time since diagnosis. In patient-completed surveys, there is the risk that not all patients provide all answers; therefore, only patients who fully completed all three questionnaires were included. Due to cross-sectional design of this study, baseline pre-treatment assessments of dysphagia, voice problems, and pain were not performed. Longitudinal research with inclusion of pre-treatment baseline values will enrich the knowledge of amount of dysphagia, voice problems, and pain in HNC patients and its course over time. Therefore, longitudinal studies are planned to be conducted in the future.

## Conclusion

The prevalence of HNC survivors is rising, and this trend is ongoing. For this reason, attention should be turned to QoL issues for HNC survivors. Previous reviews have shown that PRO surveys in cancer clinical practice can improve patient satisfaction with care and consultation outcomes [41]. Therefore, it is important to assess and monitor HNC survivors and detect the symptoms occurring during HNC and its treatment and their impact on daily functioning. The present study reveals dysphagia to be an important complication in HNC that greatly affects patients’ QoL and correlates significantly with voice problems and pain, which were reported in lower frequency. Most of the sociodemographic and behavioral factors did not predict symptom burden while clinical factors particularly correlated with dysphagia. Pain was not the predominant problem in our patient cohort; however, other studies could demonstrate that PROMs assessing cancer pain with feedback of the results to patients led to a significant reduction in pain intensity [42]. To achieve an ideal symptom control, effective patient–physician communication is critical to initiate an approach to provide the best care quality to the HNC patient.

## Data Availability

The data that support the findings of this study are available on request from the corresponding author VZ. The data are not publicly available as they contain information that could compromise research participant privacy.
